# Characterization of Butyrate-Resistant Colorectal Cancer Cell Lines and the Cytotoxicity of Anticancer Drugs against These Cells

**DOI:** 10.1155/2022/6565300

**Published:** 2022-07-19

**Authors:** Kesara Nittayaboon, Kittinun Leetanaporn, Surasak Sangkhathat, Sittiruk Roytrakul, Raphatphorn Navakanitworakul

**Affiliations:** ^1^Department of Biomedical Sciences and Biomedical Engineering, Faculty of Medicine, Prince of Songkla University, Hat Yai, Songkhla, Thailand 90110; ^2^Functional Ingredients and Food Innovation Research Group, National Center for Genetic Engineering and Biotechnology (BIOTEC), National Science and Technology Development Agency, Pathumthani 12120, Thailand

## Abstract

Colorectal cancer (CRC) is the third most common cancer worldwide. The gut microbiota plays a critical role in homeostasis and carcinogenesis. Butyrate, a short-chain fatty acid produced by the gut microbiota, plays a role in intestinal homeostasis and acts as an anticancer agent by inhibiting growth and inducing apoptosis. However, microbiota studies have revealed an abnormally high abundance of butyrate-producing bacteria in patients with CRC and indicated that it leads to chemoresistance. We characterized butyrate resistance in HCT-116 and PMF-K014 CRC cells after treatment with a maximum butyrate concentration of 3.2 mM. The 50% inhibitory concentration of butyrate was increased in butyrate-resistant (BR) cells compared with that in parental (PT) cells. The mechanism of butyrate resistance was initially investigated by determining the expression of butyrate influx- and drug efflux-related genes. We found the increased expression of influx- and efflux-related genes in BR cells compared with that in PT cells. Proteomic data showed both identical and different proteins in PT and BR cells. Further analysis revealed the crossresistance of HCT-116 cells to metformin and oxaliplatin and that of PMF-K014 cells to 5-fluorouracil. Our findings suggest that the acquisition of butyrate resistance induces the development of chemoresistance in CRC cells, which may play an important role in CRC development, treatment, and metastasis.

## 1. Introduction

Colorectal cancer (CRC) is the third most common cancer and second leading cause of cancer-related deaths worldwide. In Thailand, CRC is the third most common cancer, and 11% of the cancer-related deaths are attributed to CRC [1]. According to a World Health Organization report in 2020, the death rate associated with CRC accounted for 9% of the incidence. Several risk factors are associated with CRC, including host genetic and epigenetic alterations, dietary lifestyle, environment, and microbial community imbalance [2–5]. The gut microbiota plays a key role in homeostasis and carcinogenesis, producing metabolites to maintain intestinal barrier integrity and immune homeostasis. An imbalance in the microbiome leads to carcinogenesis [6–8]. Currently, CRC is treated using surgery and chemoradiotherapy. Conventional chemotherapy targets rapidly dividing cells and is the main treatment strategy for improving the mortality rate of patients with CRC. In Thailand, a combination of 5-fluorouracil (5-FU) and oxaliplatin (Oxa) (FuOx) is the chemotherapeutic treatment of choice for CRC [9]. The main mechanism of action of 5-FU is the inhibition of thymidylate synthase [10]. Oxa covalently binds DNA, leading to the formation of platinum-DNA adducts that induce a prolonged G2 arrest and inhibit growth, resulting in apoptotic cell death [11]. Metformin (Met) is typically used to treat diabetes mellitus (DM); however, its use is also associated with the reduction of cervical, endometrial, lung, and colon cancer risk in patients with type 2 DM [12–16]. Met inhibits mitochondrial respiration, leading to an imbalance in the AMP:ATP ratio, which is monitored by—or activates—AMP-activated protein kinase [17]. Decreased cellular invasion and increased adhesion to collagen are correlated with a reduction in cell motility in human astrocytoma (brain tumor) cell lines [18]. This information suggests the potential of Met as an anticancer drug that may kill cancer cells including butyrate-resistant (BR) CRC cells. However, currently, 90% of the chemotherapy failures occur due to the invasion and metastasis of cancer cells [19].

Butyrate, a short-chain fatty acid produced by the gut microbiota, plays a role in intestinal homeostasis by inducing the proliferation and differentiation of cells of the normal colonic epithelium. However, as an anticancer agent, butyrate inhibits the growth and induces the apoptosis of cancer cells [6, 20, 21]. Previous studies have shown that butyrate inhibits the growth of human endometrial and ovarian cancer cells [22] and induces the apoptosis of breast cancer cells [23]. However, recent microbiota studies have revealed a higher abundance of butyrate-producing bacteria in patients with CRC than in the non-CRC population [2, 24, 25]. Furthermore, a correlation exists between butyrate resistance and chemoresistance [26]. Acquired resistance to butyrate induces the development of a malignant phenotype, such as the one that decreases cell death under glucose-deprivation conditions in BR colon adenocarcinomas [27, 28]. A study on a BR CRC (HCT-116/BR) cell line indicated that resistance to butyrate resulted in the development of resistance against chemotherapies, such as paclitaxel, 5-FU, and doxorubicin [26]. Moreover, stem cell markers, such as *OCT4* and ATP-binding cassette (ABC) transporter (*ABCG2*), are also highly expressed in the HCT-116/BR cell line [29]; this may result in treatment failure. Therefore, understanding the mechanisms underlying the regulation of butyrate resistance may enable us to develop better treatment strategies to eliminate cancer cells and/or improve the quality of life of patients with CRC.

**Figure 1:**
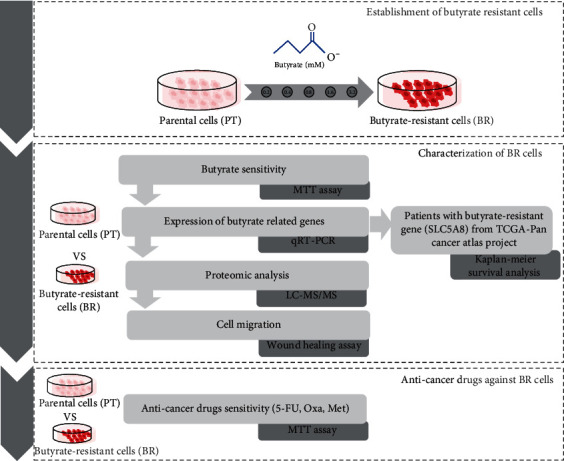
The experimental design of this study. The flow chart shows the workflow of our study.

In this study, we established a BR CRC cell line and subsequently evaluated the characteristics of the resistant cells, including cell morphology, butyrate sensitivity, and expression of butyrate- and drug efflux-related genes. Proteomic analyses were performed to determine the differences in parental (PT) and BR cells. Cell migration was used as an indicator of aggressiveness. Finally, anticancer drugs, including 5-FU, Oxa, and Met, were used to evaluate the cellular response. The experimental design is shown in Figure 1.

## 2. Materials and Methods

### 2.1. Cell Culture

The epithelial colorectal carcinoma cell lines, HCT-116 and PMF-K014, were grown in Dulbecco's Modified Eagle Medium (Gibco™ Thermo Fisher Scientific, Waltham, USA) supplemented with 10% heat-inactivated fetal bovine serum (Gibco™ Thermo Fisher Scientific) and 1% penicillin/streptomycin (Gibco™ Thermo Fisher Scientific) in a humidified incubator with an atmosphere of 5% CO_2_ at 37°C.

### 2.2. Establishment of a BR CRC Cell Line

HCT-116 and PMF-K014 cells were initially stimulated in complete-medium supplemented with 0.2 mM sodium butyrate (Sigma-Aldrich, St. Louis, USA). Butyrate treatment induced cancer cell death. However, some cells survived and continued to proliferate; these were considered BR. BR cells were subcultured till 80% confluent. Subsequently, the concentration of butyrate was increased twofold every three generations. After the concentration of butyrate reached 3.2 mM, BR cells were used in further experiments.

### 2.3. Butyrate-Sensitivity Assay

The cytotoxic effects of butyrate on the cells were determined using a tetrazolium bromide colorimetric assay (3-[4,5-dimethylthiazol-2-yl]-2,5-diphenyltetrazolium bromide; MTT). PT (HCT-PT and PMF-PT) and BR (HCT-BR and PMF-BR) cells were seeded in 96-well plates and incubated with various concentrations of butyrate (0–30 mM) for 72 h. After incubation, MTT was added to the cells and incubated for 2 h. To determine the MTT results, dimethyl sulfoxide (DMSO; Sigma-Aldrich) was added to the cells and incubated for 30 min. The absorbance of formazan was recorded using a microplate spectrophotometer system (SpectraMax 190, Molecular Devices, San Jose, USA). The results were analyzed using the SoftMax Pro software (version 2.2.1) and presented as the percentage of cell survival compared with control values.

### 2.4. Evaluation of the Expression of Butyrate-Related Genes Using Quantitative Reverse-Transcription Polymerase Chain Reaction (qRT-PCR)

To determine the expression of butyrate-related genes, total RNA was isolated from PT and BR cells using the TRIzol Reagent (Invitrogen, Waltham, USA) in accordance with the manufacturer's instructions. RNA was then quantified using absorbance measurements, 1.5% agarose gel electrophoresis, and a Nanodrop (Thermo Fisher Scientific). RNA samples (1 *μ*g) of sufficient quality were reverse-transcribed into complementary DNA (cDNA) [30, 31] using an iScript™ cDNA synthesis kit (Bio-Rad Laboratories, Hercules, USA) in accordance with the manufacturer's instructions. *GAPDH* was used as the internal control. Differential expression of butyrate-related genes and *ABC* transporters was determined. The primer sequences are listed in Table S1. Quantitative reverse-transcription PCR (qRT-PCR) was performed in duplicates with three independent experiments. The delta-delta Ct (2^-(∆∆Ct)^) method was used to calculate the relative gene expression levels.

### 2.5. Proteomic Analysis

Mass spectrometry was performed at the Functional Proteomics Technology Laboratory, National Center for Genetic Engineering and Biotechnology (BIOTEC), Thailand. The PT and BR proteins were reduced, alkylated, digested with trypsin, and analyzed by liquid chromatography-mass spectrometry (MS) (Impact II, Bruker, Billerica, USA). Differentially expressed proteins were quantified and identified using the DeCyder MS differential analysis software 2.0 (GE Healthcare, Chicago, USA) and MASCOT search engine (Matrix Science, London, UK) based on the NCBI human protein database. Proteins differentially expressed among cell types were analyzed for pathway enrichment using Kyoto Encyclopedia of Genes and Genomes (KEGG) analysis by employing *R* (version 4.1.1; *R* Foundation for Statistical Computing, Vienna, Austria). Uniquely expressed proteins in each cell type were categorized using PANTHER (version 16.0) and UniProt.

### 2.6. Cell Migration Assay

To determine the migration of each cell, cell migration or wound healing assays were conducted as previously described [32]. Briefly, a 90–95% confluent cell monolayer was scratched using a pipette tip to generate a wound. Images were captured 0, 6, 12, and 24 h after wound scratching. The ImageJ (version 1.34) was then used to calculate the wound field.

### 2.7. Anticancer Drug Sensitivity Assay

The cytotoxic effects of anticancer drugs, including 5-FU, Oxa, and Met, were evaluated using the MTT assay. PT and BR cells were seeded in 96-well plates and incubated with various concentrations of the drugs for 72 h. After incubation, MTT was added to the cells and incubated for 2 h. To detect the MTT results, DMSO (Sigma-Aldrich) was added and incubated for 30 min. The absorbance of formazan was recorded using a microplate spectrophotometer system (SpectraMax190, Molecular Devices). The results were analyzed using SoftMaxPro (version 2.2.1) and presented as the percentage of inhibition compared with control values.

### 2.8. Statistical Analysis

Data are presented as the mean ± standard deviation (SD) of at least three independent experiments. A Student's *t-*test (two-tailed, unpaired) was used to evaluate the statistical significance of results from all experiments.

## 3. Results

### 3.1. Cellular Morphology of BR Cells

**Figure 2:**
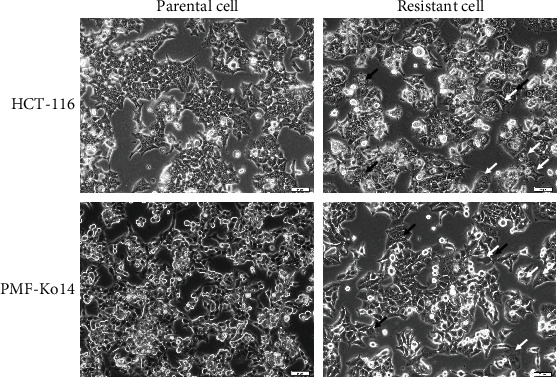
Micrographs of HCT-116 and PMF-K014 parental and butyrate-resistant subcell lines. Cell morphology was visualized using light microscopy. Increased vacuolization (black arrows) and cellular volume (white arrows) are indicated. Scale bar = 50 *μ*m.

Human colon cancer HCT-116 and PMF-K014cell lines were used to generate BR cells: HCT-BR and PMF-BR cells, respectively. To develop butyrate resistance, the cells were exposed to butyrate for three months. The morphology of the BR cells was altered slightly relative to that of the PT cells as shown in Figure 2. The HCT-116 PT cells displayed a sharp-pointed shape, while the HCT-BR cells were more rounded and expanded. The PMF-K014 PT cells displayed a polygonal epithelial structure indicating robust cell-cell interactions, while the BR cells showed a decrease in cell-cell interactions compared to the PT cells. Vacuolization was also observed in BR cells (indicated by black arrows).

### 3.2. Butyrate Sensitivity

**Figure 3:**
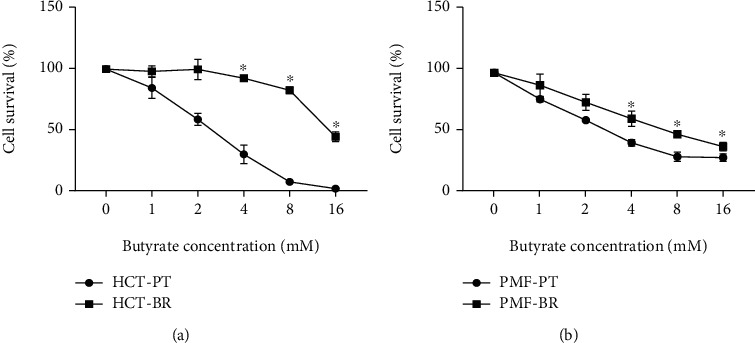
Cell survival determined using the 3-[4,5-dimethylthiazol-2-yl]-2,5-diphenyltetrazolium bromide (MTT) assay. Effects of butyrate on HCT-116 (a) and PMF-K014 (b) parental (PT) and butyrate-resistant (BR) cells. Dose-response curves of butyrate over 72 h. The data are expressed as means ± standard deviation (SD) of triplicate experiments. Statistically significant differences were determined using Student's *t*-test (^∗^*p* value<0.05). Abbreviations: HCT-PT: HCT parental cells; HCT-BR: butyrate-resistant HCT cells; PMF-PT: PMF parental cells; PMF-BR: butyrate-resistant PMF cells.

MTT assays were used to determine the 50% inhibitory concentration (IC_50_) to evaluate the butyrate sensitivity of PT and BR cells. The viability of BR cells was significantly higher than that of their PT cells. Cell survival is shown in Figure 3, and the IC_50_ of each cell line is shown in Table 1. The IC_50_ value of the HCT-BR cells was 5.38-fold higher than that of the HCT-PT cells. For PMF-BR cells, the IC_50_ was 19.72, which was 3.00-fold higher than that of their PT cells.

### 3.3. Expression of Butyrate-Related Genes and Drug Efflux Pumps

**Figure 4:**
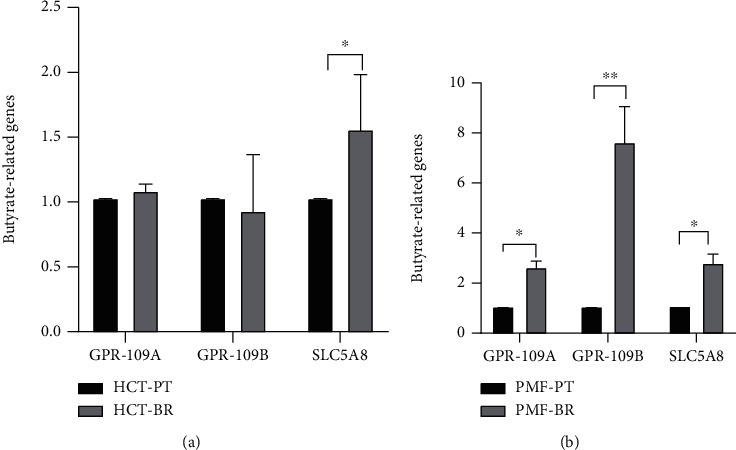
Relative expression of butyrate-related genes in HCT (a) and PMF (b) cells. Expression (*GPR109A*, *GPR109B*, and *SLC5A8*) is shown relative to that of *GAPDH*. Data are expressed as means ± SD of triplicate experiments. Significant differences were determined using Student's *t*-test (^∗^*p* value<0.05, ^∗∗^*p* value<0.01).

**Figure 5:**
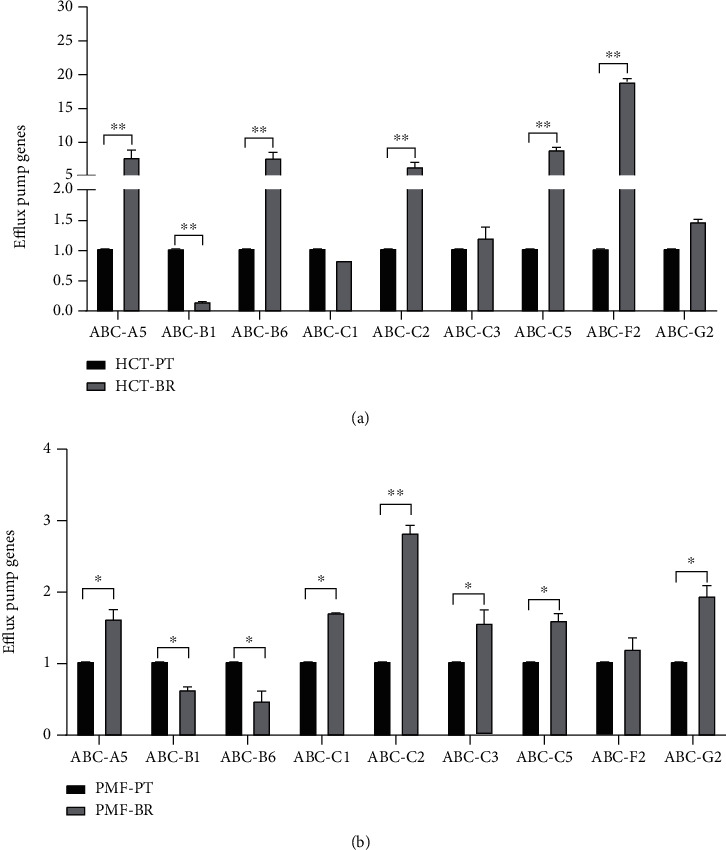
Relative expression of drug efflux genes in HCT (a) and PMF (b) cells. Expression is shown relative to *GAPDH* expression. Data are expressed as means ± SD of triplicate experiments. Significant differences were determined using Student's *t*-test (^∗^*p* value<0.05).

**Figure 6:**
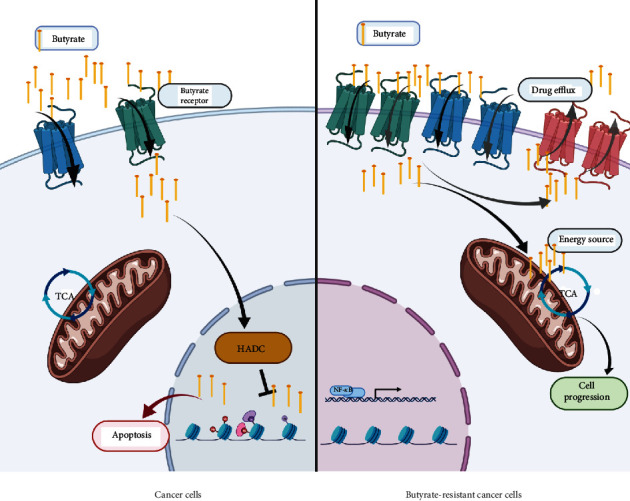
Schematic illustration of the proposed mechanism of butyrate resistance development. Abbreviations: HDAC: histone deacetylase; TCA: tricarboxylic acid cycle. The schematic was created https://usingbiorender.com/.

The expression of butyrate-related genes was determined using GAPDH as an internal control. Figures 4 and 5 show the relative expression of butyrate-related genes and drug efflux pumps, respectively. Hydroxycarboxylic acid receptor 2 (GPR109A), which is a butyrate receptor, its homolog GPR109B, and sodium-coupledmonocarboxylate transporter 1 (SLC5A8 or SMCT1) respond to the transport of bacterial metabolites, particularly butyrate, in intestinal cells. BR cells showed higher expression of these receptors than PT cells. SLC5A8 was significantly upregulated in HCT-BR cells, whereas all three receptors were significantly upregulated in PMF-BR cells. To evaluate the expression of the efflux pump, the expression of the ABC transporter, which plays an important role in drug transport in cancer cells, was evaluated. BR cells showed higher expression of this drug efflux gene than PT cells but showed different patterns of expression for the efflux genes. We found that the ABC-A5 and ABC-C5 were significantly upregulated in both BR cells compared with that in the corresponding PT cells. The expression of ABC-B1was low in both BR cell types. HCT-BR cells showed higher expression of ABC-B6, ABC-C2, ABC-C5, and ABC-F2 than HCT-PR cells. Simultaneously, PMF-BR cells showed higher expression of ABC-C1, ABC-C3, and ABC-G2 than PMF-PT cells. However, the expression of ABC-B6 was not clear. We proposed a mechanism for resistance in Figure 6.

### 3.4. Differential Protein Expression in PT and BR Cells

**Figure 7:**
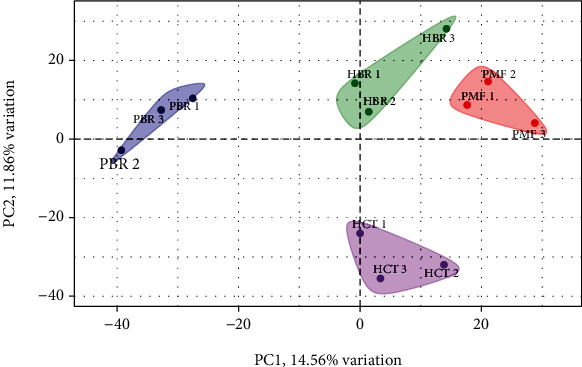
Principal component analysis (PCA) of the proteomic data in a 2-dimensionalgraph of PC1 and PC2. The biplot shows proteomic data (scores) as labeled dots and cell types as vectors for the parental (HCT and PMF) and butyrate-resistant (HBR and PBR) cells. Abbreviations: HCT: HCT parental cells; HBR: butyrate-resistant HCT cells; PMF: PMF parental cells; PBR: butyrate-resistant PMF cells.

**Figure 8:**
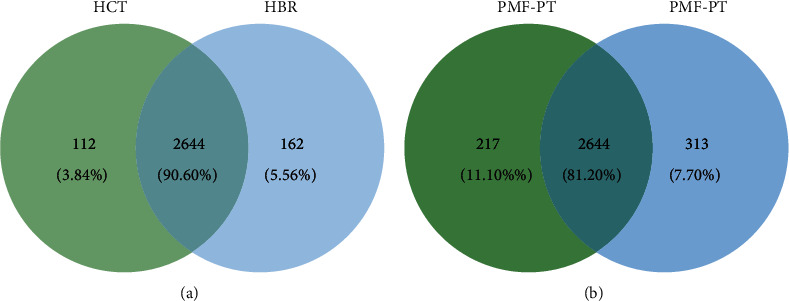
Venn diagram illustrating the relationship between HCT (a) and PMF (b) annotated genes.

**Figure 9:**
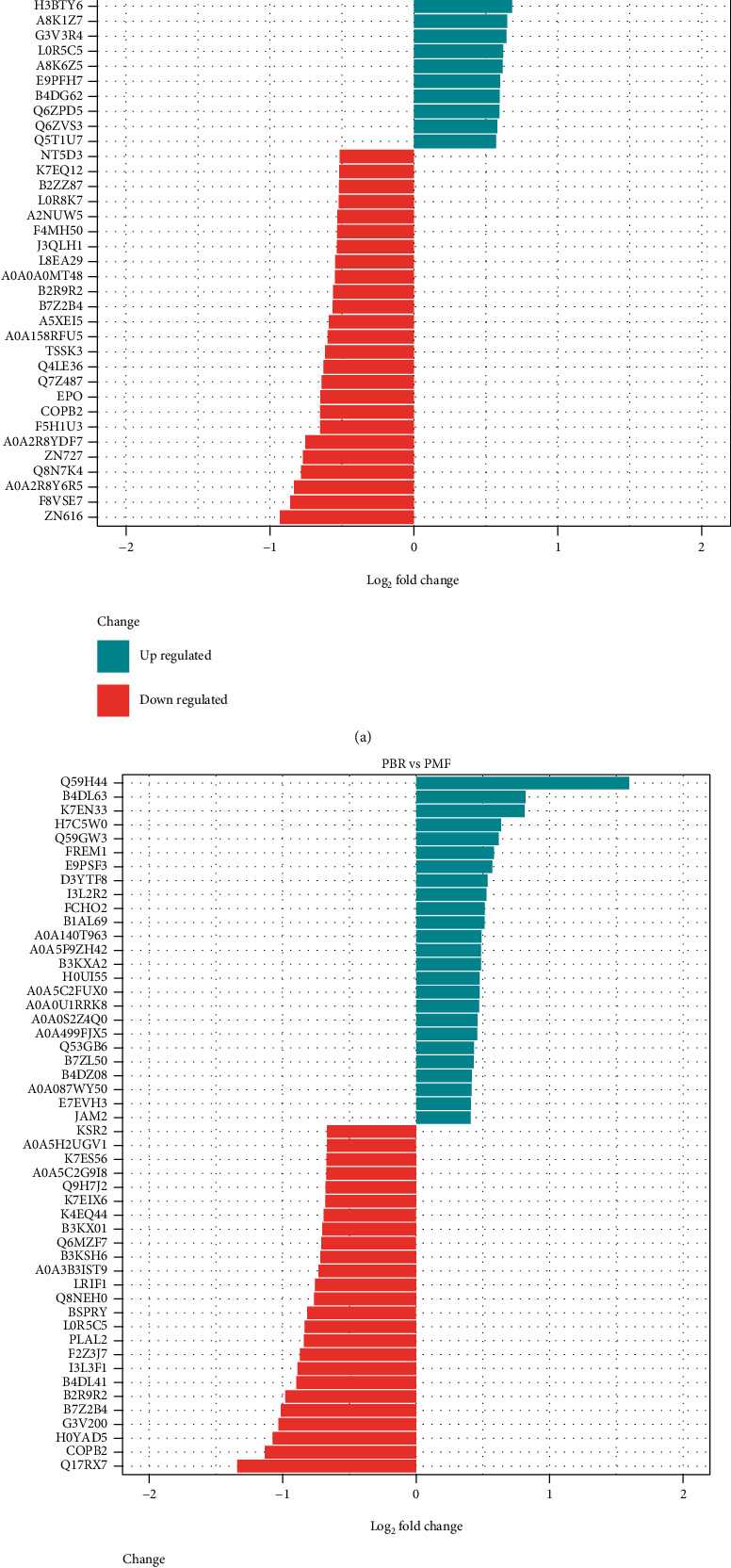
Top 10 differentially expressed proteins in HCT (a) and PMF (b) cells.

**Figure 10:**
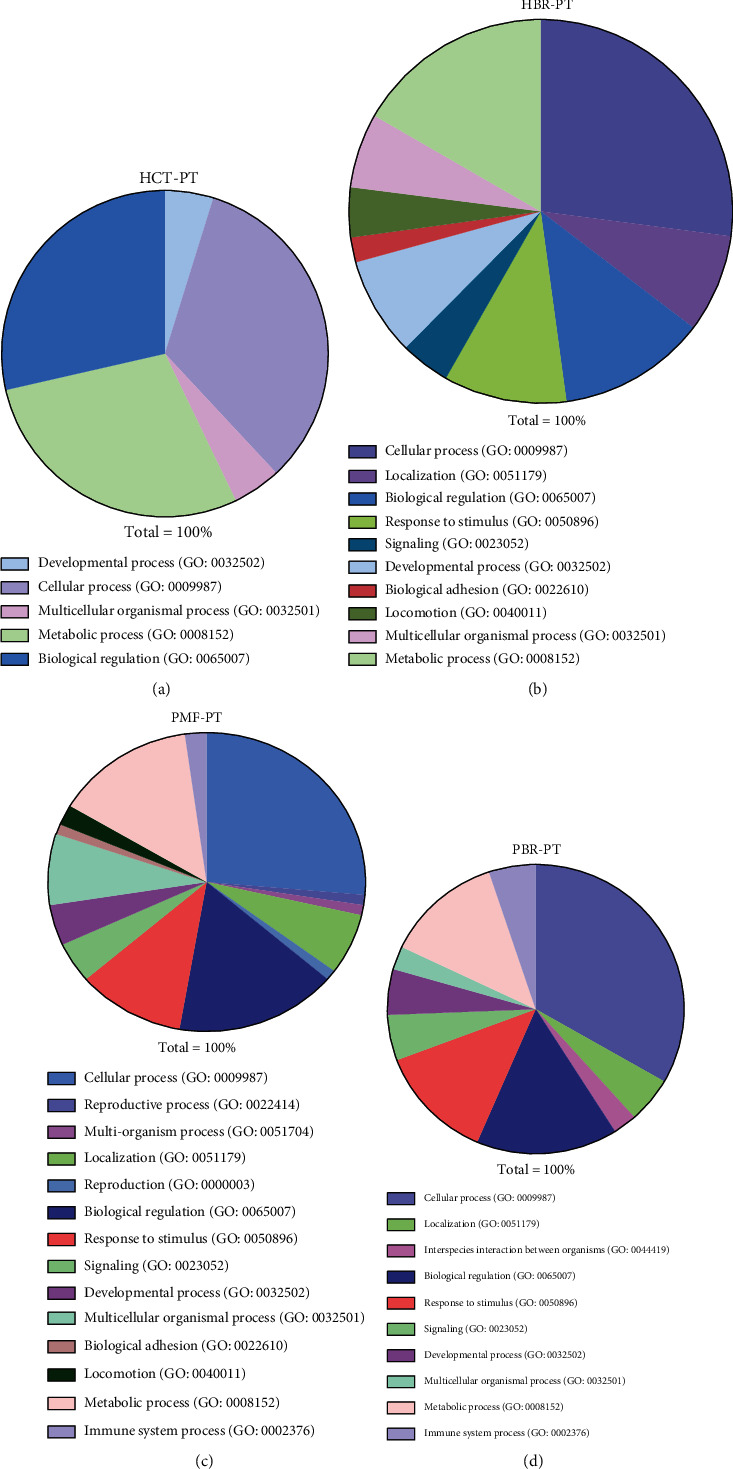
Pie chart showing the biological processes of the HCT-PT (a), HCT-BR (b), PMF-PT (c), and PMF-BR (d) cells.

**Figure 11:**
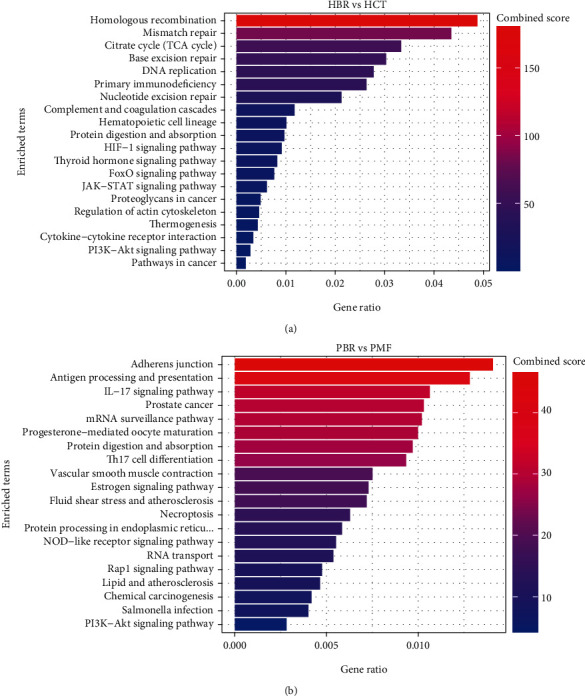
The top 20 KEGG enrichment pathways of the HCT (a) and PMF (b) cells.

To investigate the characteristics of the PT and BR cells, principal component analysis (PCA) of the proteomic data was performed (Figure 7). The low dimensional variations (PC1 14.56% and PC2 11.86%) suggested that most proteins were identical among all cells, which maybe because the principal protein characteristics of all CRC cells are the same across cell types and resistance statuses. However, some unique proteins were identified in the PCA plot and used to distinguish clusters between PT and BR cells in both cell types (i.e., butyrate-resistant PMF cells [PBR] vs. PMF in PC1 and butyrate-resistant HCT cells [HBR] vs. HCT in PC2). Venn diagrams (Figure 8) confirmed this finding by showing that PT and BR cells shared 90% (2,644 proteins) and 80% (2,285 proteins) of the proteins in HCT and PMF cells, respectively. We analyzed the unique proteins according to cell type and resistance status and found that 3.84% (112 proteins) and 5.56% (162 proteins) were found in HCT-PT and HCT-BR cells, respectively. Unique proteins in PMF cells accounted for 11.10% (313 proteins) and 7.70% (217 proteins) in PT and BR cells, respectively. The differential expression of proteins is shown in Figure 9, and the protein names are listed in Tables 2 and 3. We identified differential expression of proteins between the HCT and PMF cells, and 2,916 and 2,816 proteins were differentially expressed in HCT and PMF cells, respectively. Notably, the expression of coatomer complex subunit beta 2(COPB2), an essential protein required for Golgi budding and vesicular trafficking [33], was decreased in both HCT-BR and PMF-BR cells, compared with that in the corresponding PT cells. A pie chart showing the biological process annotations of the proteins in each cell type is shown in Figure 10. HCT-BR cells expressed more biological processes than their PT cells did, including localization, response to stimuli, signaling, biological adhesion, and locomotion. For the PMF cells, PMF-BR expressed fewer biological processes than their PT cells did, except the interspecies interaction between organisms, with PMF-PT cells expressing greater reproductive and multiorganism processes, reproduction, biological adhesion, and locomotion. We then used a bar graph to illustrate KEGG pathway enrichment (Figure 11). The pathways that were enriched in both PT cell lines were protein digestion and absorption, the phosphatidylinositol 3-kinase/Akt pathway, and pathways involved in cancers. For HCT-BR cells, pathways involved in homologous recombination and mismatch repair and signaling pathways were enriched. For PMF-BR cells, adherent junctions, cytokines, and receptors were enriched.

### 3.5. Cell Migration

**Figure 12:**
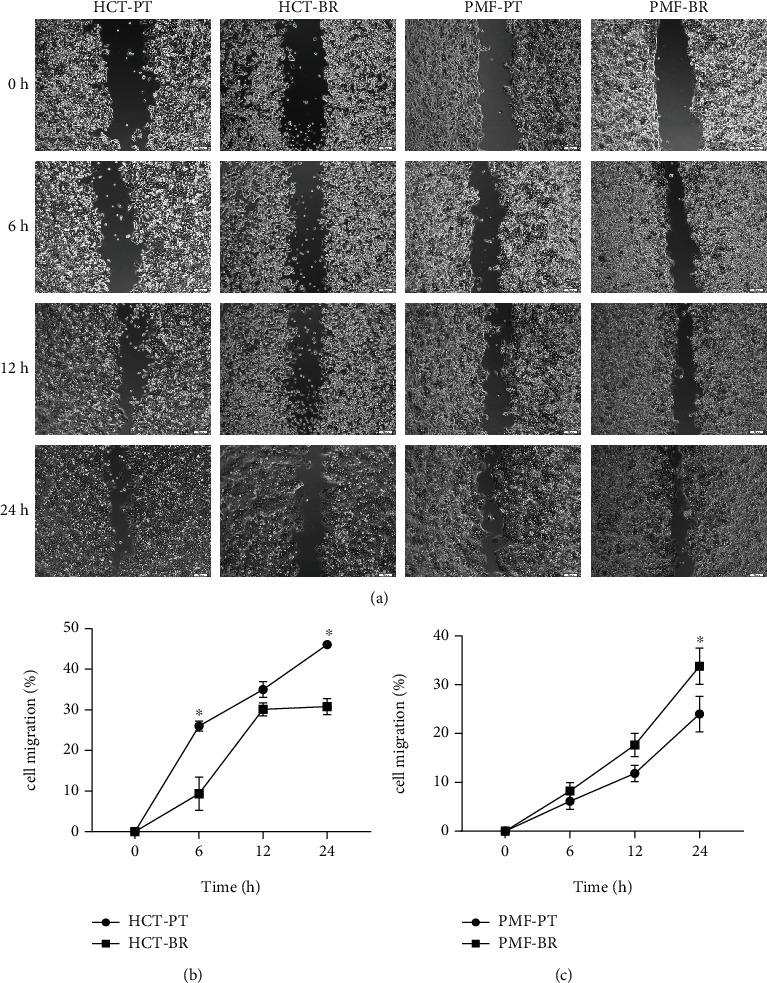
The capacity for cell migration (%) determined by wound healing assays. Parental and butyrate-resistant cells were incubated 24 h after wound field generation. (a) Wound fields were captured by inverted microscopy (×10 magnification). Scale bar = 100 *μ*m. The line graph represents the percentage of migration of HCT (b) and PMF (c) cells. Significant differences were determined using Student's *t*-test (^∗^*p* value <0.05).

To examine the migration capacity of PT and BR cells, we conducted wound healing assays. The field containing the wound area after 24 h is shown in Figure 12, and the percentage of migrated cells is shown in Figure 12(b)and Table S2. We found a significant difference between the migration rates of PT and BR cells. The migration rate in HCT-PT cells (45.81%) was higher than in BR cells(33.29%). However, the migration rate in PMF-BR cells (37.96%)was higher than in their PT cells (25.39%).

### 3.6. Anticancer Drug Sensitivity

To test the anticancer drug sensitivity of the cells, the MTT results were used to calculate the IC_50_. The IC_50_ of each cell was determined using data from three independent experiments and analyzed using Student's t-test. The IC_50_ values of PT and BR cells are listed in Table 4. We found crossresistance of chemotherapy agents in BR cells; HCT-BR cells showed crossresistance to Oxa at 28.67 *μ*M (13.5-fold) and MET at 6.41 mM (3.66-fold). However, PMF-BR cells showed crossresistance to 5-FU at 26.18 *μ*M (1.7-fold).

## 4. Discussion

Butyrate is an anticancer agent against which resistance has been reported in CRC cells. However, the mechanism underlying the development of butyrate resistance in CRC cells remains unclear. Investigation of the mechanism by which butyrate resistance develops in cells may help establish new therapeutic strategies for CRC. HCT-BR and PMF-BR cells were established by continuously exposing the cells to butyrate. BR cells showed slight differences in morphology with respect to their PT cells based on the results of inverted light microscopy. Increasing vascularization and cellular volume were found in BR cells. This result is similar to that of previous studies in BCS-TC2. BR2 cells, a BR human colon adenocarcinoma cell line, showed an increase in vacuolization and cellular volume [27]. The butyrate sensitivity test revealed that BR cells had a higher survival rate than the PT cells in both cell lines. The IC_50_ values were also higher in BR cells than in PT cells in both cell lines. These results confirm that the BR cells exhibit greater resistance to butyrate than PT cells.

Several mechanisms are involved in the resistance to butyrate, including alteration of the drug target and drug inactivation and efflux. To investigate the underlying mechanism in BR cells, we evaluated the expression of butyrate-related genes, including the butyrate receptors, *GPR109A* and *GPR109B*, and butyrate transporter, *SLC5A8*. We found different patterns of expression of butyrate-related genes in the two cell lines. HCT-BR cells showed an upregulation of *SLC5A8*, while PMF-BR cells showed an upregulation of *GPR109A*, *GPR109B*, and *SLC5A8*. In a previous study, the expression of butyrate-related genes was upregulated in BR mice compared with that in germ-free mice [34]. We also performed Kaplan–Meier survival analysis to evaluate the correlation between butyrate-related gene expression and survival outcome of patients with cancer treated with 5-FU and/or Oxa using The Cancer Genome Atlas (TCGA) pan-cancer data portal. Interestingly, the Kaplan–Meier analysis showed that only patients with CRC with high *SLC5A8* expression displayed significantly worse survival than patients with low expression (Figure S1). This finding suggests that high *SLC5A8* expression found in BR cells represents a lower survival outcome in patients with CRC. We further investigated drug efflux gene expression. We found that the expression of *ABC-B6*, *ABC-C5*, and *ABC-F2* was upregulated in HCT-BR cells, whereas that of *ABC-C1*, *ABC-C3*, and *ABC-G2* was upregulated in PMF-BR cells. Moreover, *ABC-A5* and *ABC-C2* were upregulated in both BR cells. *ABC-C1* and *ABC-G2* are upregulated in Hep2-5-FU-resistant cells [35]. These two *ABC* transporters are considered chemoresistance-driving genes and play a role in the acquisition of chemoresistance. Additionally, *ABC-B6*, *ABC-C1*, *ABC-C3*, *ABC-C5*, *ABC-C10*, and *ABC-F2* are found in paclitaxel-resistant cells. Furthermore, doxorubicin resistance in breast cancer cells is induced by the overexpression of *ABC-C1* and *ABC-F2* [36, 37]. Lastly, cells expressing *ABC-A5* and *ABC-F2* show stem cell features [38]. We further evaluated the cytotoxicity of anticancer drugs including Met, 5-FU, and Oxa against PT and BR cells in both HCT and PMF cells. The results revealed different drug responses in different cells. HCT-BR showed a crossresistance toward Met and Oxa, while PMF-BR showed a crossresistance to 5-FU; similarly, our previous study demonstrated that PMF-BR spheroid cells were also crossresistant to 5-FU [39]. This crossresistance may be due to the upregulation of *ABC* transporters.

Proteomic analysis identified differential expression of proteins in HCT and PMF cells. PCA showed a difference between the BR and PT cell groups. A 14.56% variation in PC1 showed a shared dimension in HCT cells but presented a dissimilarity in PMF cells. Simultaneously, an 11.86% variation in PC2 showed a shared dimension in PMF cells whereas indicated a distinct feature of HCT cells. PCA indicated that there were some differences and similarities between PT and BR cells, and the Venn diagrams correlated with these results. The Venn diagram showed that several proteins were shared between the PT and BR cells; however, there were some unique proteins present in each cell, which resulted in differences in cell characteristics. We used the University of Alabama at Birmingham Cancer (UALCAN) data analysis portal (http://ualcan.path.uab.edu/index.html) to explore the relevant proteins enriched for the differentially expressed genes. We found that the expression of COPB2 was decreased in both HCT-BR and PMF-BR cells. Regarding this unique finding in HCT-BR cells, we discovered two proteins from UALCAN that showed increased expression levels: xanthine dehydrogenase (XDH) and formin homology 2 domain-containing protein 1 (FHDC1). In the PMF-BR cells, the expression of FRAS1-related extracellular matrix 1 (FREM1) showed an increase. The COPB2 protein is related to a dilated endoplasmic reticulum (ER) with granular material, prominent rough ER, and vacuoles resulting in intracellular trafficking deficiency [33]. In cancer, COPB2 is highly expressed in glioblastomas and hepatocellular carcinomas, resulting in a worse overall survival [40, 41]. Additionally, studies on CRC cells have found that COPB2 plays an essential role in cancer cell proliferation and cell cycle progression [42]. The UALCAN database showed that the expression of COPB2 increases in CRC patients. However, the levels of the phosphorylated form of this protein were found to be decreased in every stage of CRC (Figure S2). The level and function of COPB2 across different cancers is controversial. Therefore, the function of COPB2 in BR-CRC requires further investigation. In HCT-BR cells, XDH exerts purine oxidation and electron acceptor functions and is highly expressed in uterine corpus endometrial carcinoma and lung and pancreatic cancers(Figure S3). Furthermore, XDH is expressed at lower levels in colon adenocarcinoma tissue than in healthy tissue. Our study was performed in HCT cells which are derived from primary colorectal carcinoma [43]. Therefore, there may be a difference in the expression of XDH. A previous pan-cancer study showed that XDH is involved in proinflammatory and immune stimulation. Additionally, increases in XDH combined with adenine phosphoribosyl transferase, a key enzyme in the purine salvage pathway, result in increased sensitivity to 5-FU [44]. This finding requires further investigation to predict the effects of 5-FU more effectively in patients with CRC and the BR phenotype. Another upregulated protein in HCT-BR cells was FHDC1, a microtubule-associated formin involved in regulating actin and microtubule dynamics. The expression of FHDC1 is increased in various cancers including CRC and at its highest level in stage 1 cancer (Figure S4). A study in lung adenocarcinoma reported that the high expression of FHDC1 was associated with significantly improved survival outcomes compared with that of low expression [45]. In PMF polygonal epithelial cells derived from the peritoneal dissemination of highly metastatic patients with CRC [46], we found the higher expression of FREM1 in CRC patients than in healthy patients, with the highest expression in stage 4 patients (Figure S5). FREM1 plays a role in epidermal differentiation and is required for epidermal adhesion during embryonic development. However, the function of FREM1 in cancer is not well understood. A study in breast cancer showed that the expression of FREM1 was dramatically decreased, which correlated with a lower overall and recurrence-free survival [47]. However, the expression of FREM1 in CRC differs from that in breast cancer. Moreover, FREM1 levels increased in the primary tumor and higher cancer stages. However, the function of FREM1 in CRC remains unclear and requires investigation.

Cell migration was also investigated to evaluate the aggressiveness of BR and PT cells, and differences were found. HCT-BR showed a lower migration rate than HCT-PT. This may be due to the downregulation of the erythropoietin receptor, which is related to the downregulation of the JAK2/STAT5 signaling cascade [48, 49]. In contrast, the PMF-BR cells showed a higher migration rate than PMF-PT cells. The protein expression profile showed the upregulation of Notchless protein homolog 1 in PMF-BR cells. This protein plays a role in Notch activity which is known to promote cell migration and invasion in brain cancer [50].

Taken together, our findings suggest that 5-FU may be used as a potential anticancer agent in HCT-BR cells, representing primary tumor treatment. Meanwhile, Met and Oxa may be effective in PMF-BR cells, which represent metastatic tumors. However, further studies are needed to investigate the effects of Met.

## 5. Conclusions

Herein, we investigated the characteristics of butyrate resistance in CRC cells in both primary and metastatic conditions. The mechanisms underlying the development of butyrate resistance involve an increase in the expression levels of various efflux genes. The proteins expressed in the BR cells shared some parental characteristics, while unique proteins showed characteristic of resistance. Our study also confirmed that chemotherapy resistance arises from butyrate resistance in CRC cells. Moreover, Met showed a potential therapeutic effect against PMF-BR cells. Thus, further investigation of Metin, the molecular mechanism underlying butyrate resistance, is needed for its use in clinical settings to enhance the effectiveness of CRC therapies. Lastly, the effects of anticancer drugs on BR CRC cells requires further investigation in animal models.

## Figures and Tables

**Table 1:** IC_50_ values of butyrate according to the cells.

Cell lines	Butyrate (mM, mean ± SD)	Fold	*p* value (*t*-test)
HCT-PT	2.76 ± 0.05	5.38	>0.01
HCT-BR	14.85 ± 0.67		
PMF-PT	6.57 ± 0.80	3.00	>0.01
PMF-BR	19.72 ± 1.62		

**Table 2:** Top 10 upregulated and downregulated proteins in HCT cells.

HBR vs. HCT	Entry	Protein	Function
Up	XDH	Xanthine dehydrogenase/oxidase	Key enzyme in purine degradation
	B7Z1X3	Dynein regulatory complex subunit 4	Microtubule binding, small GTPase binding
	H0Y9Y8	RUN and FYVE domain-containing protein 1	Binds phospholipid vesicles containing phosphatidylinositol 3-phosphate and participates in early endosomal trafficking
	A0A2R8Y734	Thrombopoietin	Lineage-specific cytokine affecting the proliferation and maturation of megakaryocytes from their committed progenitor cells
	A8MXR8	PHD finger protein 20-like protein 1	Regulation of transcription, DNA-templated
	H7BXL6	Otogelin-like protein	Extracellular region
	CCKAR	Cholecystokinin receptor type A	Receptor for cholecystokinin. Mediates pancreatic growth and enzyme secretion, and smooth muscle contraction of the gall bladder and stomach.
	PRAM	PML-RARA-regulated adapter molecule 1	Lipid binding, protein kinase binding, and integrin-mediated signaling pathway
	FHDC1	FH2 domain-containing protein 1	Protein localization to plasma membrane
	A0A024RDL0	DNA-directed RNA polymerase III subunit RPC9	Microtubule-associated formin which regulates both actin and microtubule dynamics

Down	Q7Z487	Transforming growth factor beta 1	DNA-directed 5′-3′RNA polymerase activity and nucleotide binding
	EPO	Erythropoietin receptor	Growth factor activity
	COPB2	Coatomer subunit beta′	Receptor for erythropoietin mediates erythropoietin-induced erythroblast proliferation and differentiation. Upon EPO stimulation, EPOR dimerizes triggering the JAK2/STAT5 signaling cascade
	F5H1U3	Peptidylprolyl isomerase	The coatomer is a cytosolic protein complex that binds to dilysine motifs and reversibly associates with Golgi nonclathrin-coated vesicles, which further mediate biosynthetic protein transport from the ER, via the Golgi up to the trans Golgi network
	A0A2R8YDF7	Lysine-specific demethylase 4A	FK506 binding
	ZN727	Putative zinc finger protein 727	Heat shock protein binding
	Q8N7A4	cDNA FLJ25865 cis, clone CBR01927	Peptidyl-prolyl cis-transisomerase activity
	A0A2R8Y6R5	Caseinolytic peptidase B protein homolog	Histone demethylase that specifically demethylates “Lys-9” and “Lys-36” residues of histone H3, thereby playing a central role in the histone code
	F8VSE7	Transcription factor E2F7	DNA-binding transcription factor activity, RNA polymerase II-specific
	ZN616	Zinc finger protein 616	Uncharacterized protein

Abbreviations: HCT: HCT parental cells; HBR: butyrate-resistant HCT cells.

**Table 3:** Top 10 upregulated and downregulated proteins in PMF cells.

PBR vs. PMF	Entry	Protein	Function
Up	Q59H44	Lymphocyte antigen 75 variant	Integral component of membrane
	B4DL63	cDNA FLJ51231, highly similar to mitochondrial ornithine transporter 1	Integral component of membrane
	K7EN33	Notchless protein homolog 1	Plays a role in regulating notch activity. Plays a role in regulating the expression of CDKN1A and several members of the Wnt pathway, probably via its effects on notch activity
	H7C5W0	DnaJ homolog subfamily B member 5	Chaperone binding, unfolded protein binding
	Q59GW3	ST8 alpha-N-acetyl-neuraminide alpha-2,8-sialyltransferase 3 variant	Sialyltransferase activity, protein glycosylation
	FREM1	FRAS1-related extracellular matrix protein 1	Extracellular matrix protein that plays a role in epidermal differentiation and is required for epidermal adhesion during embryonic development
	E9PSF3	Bromodomain and PHD finger-containing protein 3	Metal ion binding
	D3YTF8	Thioredoxin-disulfide reductase	Protein has several cofactor binding sites
	I3L2R2	Protein PIMREG	During mitosis, may play a role in the control of metaphase-to-anaphase transition
	FCHO2	F-BAR domain only protein 2	Functions in an early step of clathrin-mediated endocytosis. Has both a membrane binding/bending activity and the ability to recruit proteins essential to the formation of functional clathrin-coated pits.

Down	PLAL2	Zinc finger protein PLAGL2	DNA-binding transcription activator activity, RNA polymerase II-specific, lipid metabolic process, positive regulation of intrinsic apoptotic signaling pathway
	F2Z3J7	Rab-like protein 2B	GTPase activity, GTP binding
	I3L3F1	Caspase recruitment domain-containing protein 14	Acts as a scaffolding protein that can activate the inflammatory transcription factor NF-kappa-B and p38/JNK MAP kinase signaling pathways
	B4DL41	cDNA FLJ57825, highly similar to DNA-dependent protein kinase catalytic subunit	Kinase activity, molecular function: kinase, transferase
	B2R9R2	cDNA, FLJ94517, highly similar to Homo sapiens baculoviral IAP repeat-containing 4 (BIRC4), mRNA	Metal ion binding
	B7Z2B4	cDNA FLJ53389, highly similar to Homo sapiens RAB GTPase activating protein 1 (RABGAP1), mRNA	GTPase activator activity
	G3V200	Liprin-alpha-2	Alters PTPRF cellular localization and induces PTPRF clustering. May regulate the disassembly of focal adhesions. May localize receptor-like tyrosine phosphatases type 2A at specific sites on the plasma membrane
	H0YAD5	Probable ATP-dependent RNA helicase DDX46	Protein predicted
	COPB2	Coatomer subunit beta 2	The coatomer is a cytosolic protein complex that binds to dilysine motifs and reversibly associates with Golgi nonclathrin-coated vesicles, which further mediate biosynthetic protein transport from the ER, via the Golgi up to the trans Golgi network.
	Q17RX7	Ras association (RalGDS/AF-6) domain family member 1	Intracellular signal transduction

Abbreviations: PMF: PMF parental cells; PBR: butyrate-resistant PMF cells.

**Table 4:** The cytotoxicity values (IC_50_) of the anticancer agents against the HCT and PMF cell lines.

Cell lines	Metformin (mM, mean ± SD)	Fluorouracil (*μ*M, mean ± SD)	Oxaliplatin (*μ*M mean ± SD)
HCT-PT	1.75 ± 0.07	9.70 ± 0.09	2.13 ± 0.13
HCT-BR	6.41 ± 0.18^∗∗^	9.45 ± 0.27	28.76 ± 3.43^∗∗^
PMF-PT	1.67 ± 0.28	15.07 ± 1.74	28.15 ± 3.90
PMF-BR	1.58 ± 0.15	26.18 ± 4.37^∗^	26.92 ± 4.82

^∗^p value <0.05, ^∗∗^p value <0.01.

## Data Availability

The data that support the findings of this study are available from the corresponding author (Navakanitworakul, R), upon reasonable request (nraphatp@medicine.psu.ac.th).

## Supplementary Materials

Supplementary Materials Supplementary 1. Table S1: primer sequences used for butyrate receptors and drug efflux pumps. Supplementary 2. Table S2: the migration rate (%) of parental and butyrate-resistant cells. Figure S1: Kaplan–Meier survival analysis of cancer patients treated with 5-fluorouracil (5-FU), oxaliplatin (Oxa), or metformin (Met). (a)–(d) The survival probability curve related to SLC5A8 expression in various cancer types, such as colon adenocarcinoma (COAD) and rectal adenocarcinoma (READ) (a), stomach adenocarcinoma (STAD) (b), breast invasive carcinoma (BRCA) (c), and pancreatic adenocarcinoma (PAAD) (d). Abbreviations: TCGA: The Cancer Genome Atlas. Supplementary 4. Figure S2: the expression level of coatomer complex subunit beta (COPB2) in various types of cancers. (a) COPB2 showed higher expression in cancer patients than in healthy tissue. (b) Comparison between the expression of COPB2 in colorectal cancer in healthy and primary tumor tissues. (C) The expression of the phosphorylated form of COPB2 in each stage of cancer. *Z* values represent standard deviations from the median across samples for the given cancer type. Abbreviations: CPTAC: Clinical Proteomic Tumor Analysis Consortium; RCC: renal cell carcinoma; UCEC: uterine corpus endometrial carcinoma. Figure S2: expression of coatomer complex subunit beta 2 (COPB2) in various types of cancers. (a) COPB2 showed higher expression in cancer patients than in healthy individuals. (b) Comparison between the expression of COPB2 in colorectal cancer in healthy and primary tumor tissues. (c) Levels of the phosphorylated form of COPB2 in each stage of cancer. *Z* values represent standard deviations (SD) from the median across samples for the given cancer type. Abbreviations: CPTAC: Clinical Proteomic Tumor Analysis Consortium; RCC: renal cell carcinoma; UCEC: uterine corpus endometrial carcinoma. Figure S3: expression of xanthine dehydrogenase (XDH) in various types of cancers. (a) XDH showed higher expression in cancer patients than in normal healthy individuals. (b) Expression of XDH in colorectal cancer in normal healthy and primary tumor tissues. (c) Expression of XDH in each stage of cancer. *Z* values represent SD from the median across samples for the given cancer type. Figure S4: expression of formin homology 2 domain-containing protein 1 (FHDC1) in various types of cancers. (a) FHDC1 showed higher expression in cancer patients than in normal healthy individuals. (b) Expression of FHDC1 in colorectal cancer in normal healthy and primary tumor tissues. (c) Expression of FHDC1 in each stage of cancer. Log2 spectral count ratio values from CPTAC were first normalized within each sample profile and then normalized across samples. Figure S5: expression of FRAS1-related extracellular matrix 1 (FREM1) in various types of cancers. (a) FREM1 showed higher expression in cancer patients than in normal healthy individuals. (b) Expression of FREM1 in colorectal cancer in normal healthy and primary tumor tissues. (c) Expression of FREM1 in each stage of cancer. Log2 spectral count ratio values from CPTAC were first normalized within each sample profile and then normalized across samples.
