# Supplementary material for: Characterization of Butyrate-Resistant Colorectal Cancer Cell Lines and the Cytotoxicity of Anticancer Drugs against These Cells
Source: Biomed Res Int. 2022 Jul 19;2022:6565300. doi: 10.1155/2022/6565300 (PMC9325644; doi:10.1155/2022/6565300)
Supplement: Supplementary Materials — Supplementary 1. Table S1: primer sequences used for butyrate receptors and drug efflux pumps. Supplementary 2. Table S2: the migration rate (%) of parental and butyrate-resistant cells. Figure S1: Kaplan–Meier survival analysis of cancer patients treated with 5-fluorouracil (5-FU), oxaliplatin (Oxa), or metformin (Met). (a)–(d) The survival probability curve related to SLC5A8 expression in various cancer types, such as colon adenocarcinoma (COAD) and rectal adenocarcinoma (READ) (a), stomach adenocarcinoma (STAD) (b), breast invasive carcinoma (BRCA) (c), and pancreatic adenocarcinoma (PAAD) (d). Abbreviations: TCGA: The Cancer Genome Atlas. Supplementary 4. Figure S2: the expression level of coatomer complex subunit beta (COPB2) in various types of cancers. (a) COPB2 showed higher expression in cancer patients than in healthy tissue. (b) Comparison between the expression of COPB2 in colorectal cancer in healthy and primary tumor tissues. (C) The expression of the phosphorylated form of COPB2 in each stage of cancer. Z values represent standard deviations from the median across samples for the given cancer type. Abbreviations: CPTAC: Clinical Proteomic Tumor Analysis Consortium; RCC: renal cell carcinoma; UCEC: uterine corpus endometrial carcinoma. Figure S2: expression of coatomer complex subunit beta 2 (COPB2) in various types of cancers. (a) COPB2 showed higher expression in cancer patients than in healthy individuals. (b) Comparison between the expression of COPB2 in colorectal cancer in healthy and primary tumor tissues. (c) Levels of the phosphorylated form of COPB2 in each stage of cancer. Z values represent standard deviations (SD) from the median across samples for the given cancer type. Abbreviations: CPTAC: Clinical Proteomic Tumor Analysis Consortium; RCC: renal cell carcinoma; UCEC: uterine corpus endometrial carcinoma. Figure S3: expression of xanthine dehydrogenase (XDH) in various types of cancers. (a) XDH showed higher expression in cancer patients [file 6565300.f1.docx]

**Table S1**. Primer sequences used for amplifying butyrate receptors and drug efflux pumps.

| **Name** | **Sequence (5'-3')** | **Size (bp)** | **Note** |
| --- | --- | --- | --- |
| GAPDH-F | ACG GAT TTG GTC GTA TTG G | 207 | Housekeeping gene |
| GAPDH-R | GGA AGA TGG TGA TGG GAT TT |  |  |
| GPR109A-F | GGA CAA CTA TGT GAG GCG TTG G | 650 | Butyrate receptors |
| GPR109A-R | GGG CTG GAG AAG TAG TAC ACC |  |  |
| GPR109B-F | CGT GAT GGA CTA TGT GCG | 280 |  |
| GPR109B-R | ATT TGC AGG GCC ATT CTG GAT |  |  |
| SLC5A8-F | GGG TGG TCT GCA CAT TCT ACT | 351 |  |
| SLC5A8-R | GCC CAC AAG GTT GAC ATA GAG |  |  |
| ABC-A5-F | ATC ATG TGA GGC TGC TCA G | 149 | Drug efflux pumps |
| ABC-A5-R | ACA GCA GTT TCT CCC ATA |  |  |
| ABC-B1-F | GAA ACC AAC TGT CAG TGT ATT TTC | 110 |  |
| ABC-B1-R | AGA GGA AGT CCA GCC CC |  |  |
| ABC-B6-F | CCT GGT GTT CAA TGT CAT CC | 168 |  |
| ABC-B6-R | CAT AGC ACG AAA CTT GG |  |  |
| ABC-C1-F | TCA GCC AGA AAA TCC AC | 196 |  |
| ABC-C1-R | GGC ACC ATG AGG ACC ATC |  |  |
| ABC-C2-F | TCC TGG TTG ATG AAG GCT C | 186 |  |
| ABC-C2-R | CAG TGA ATA AGA GGA TTG CAC A |  |  |
| ABC-C3-F | GCC ACC CTG ATA CAG T | 141 |  |
| ABC-C3-R | AGA TCT CAC CCT CTG CCT TG |  |  |
| ABC-C5-F | TGA GGG AGA GAA CCA GCA CT | 229 |  |
| ABC-C5-R | AAG TAG TCC GGA TGG GCT TC |  |  |
| ABC-C10-F | GTT GGA ACC AGC CTG GAG | 232 |  |
| ABC-C10-R | CCA AAG AGG ATG TTG TCT CG |  |  |
| ABC-F2-F | GTC TCC CAT TCC CAG GAT TT | 182 |  |
| ABC-F2-R | CTG ATC TTG CTC CCA GTG AA |  |  |
| ABC-G2-F | AGC AGG TCA GAG TGT GG | 280 |  |
| ABC-G2-R | GAT CGA TGC CCT GCT TTA CC |  |  |

**Table S2**. The migration rate (%) of parental and butyrate-resistant cells.

| Cell lines | Average migration rate (%, mean ± SD) | | | |
| --- | --- | --- | --- | --- |
|  | 0 h | 6 h | 12 h | 24 h |
| HCT-PT | 0 | 26.32 ± 1.76 | 26.39 ± 0.26 | 45.81 ± 8.01 |
| HCT-BR | 0 | 7.07 ± 2.86 | 24.26 ± 1.25 | 33.29 ± 11.05 |
| PMF-PT | 0 | 6.20 ± 2.39 | 13.01 ± 6.23 | 25.39 ± 5.95 |
| PMF-BR | 0 | 6.99 ± 0.73 | 15.73 ± 0.71 | 37.96 ± 4.90 |


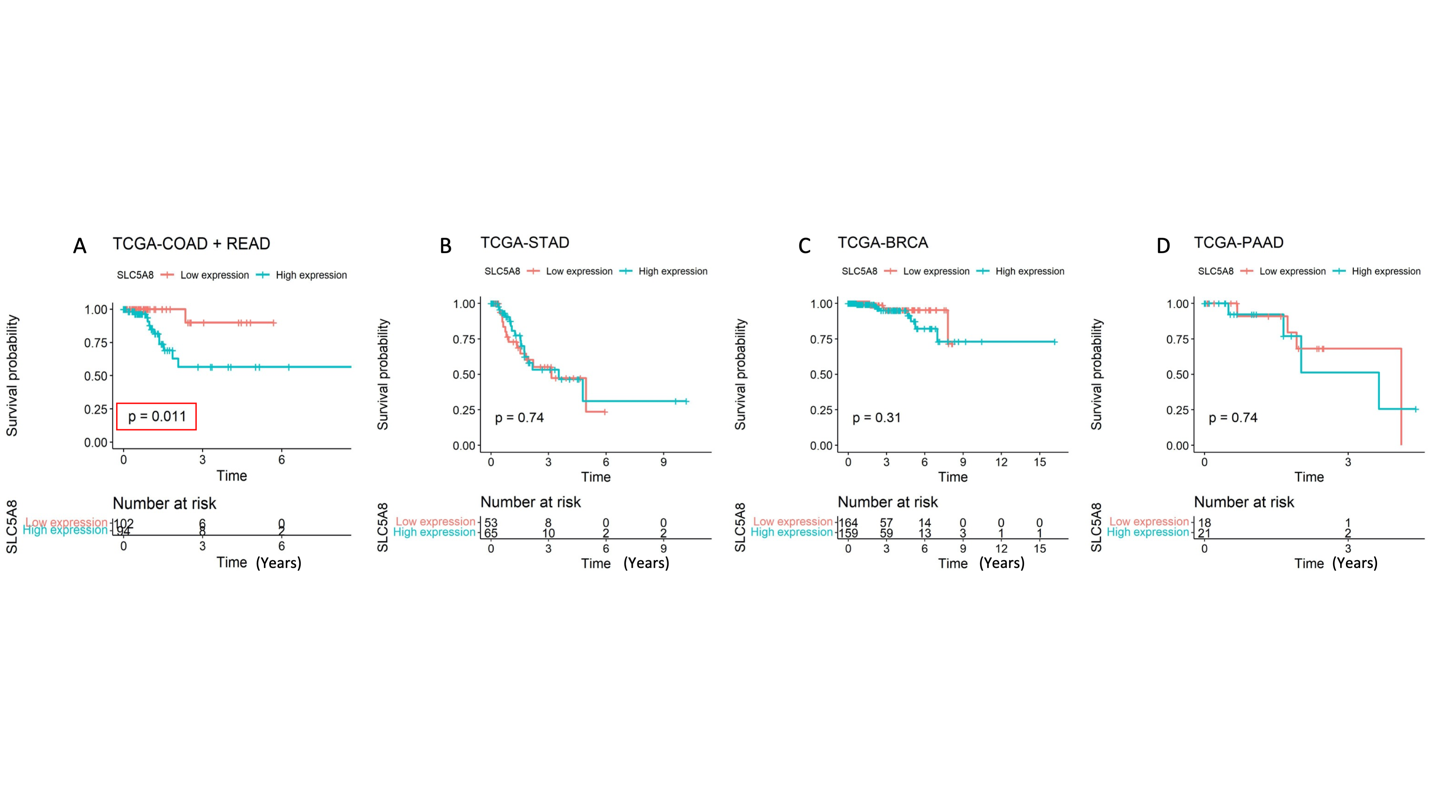


**Figure S1**. Kaplan‒Meier survival analysis of cancer patients treated with 5-fluorouracil (5-FU), oxaliplatin (Oxa), or metformin (Met). (A-D) The survival probability curve related to *SLC5A8* expression in various cancer types, such as colon adenocarcinoma (COAD) and rectal adenocarcinoma (READ) (A), stomach adenocarcinoma (STAD) (B), breast invasive carcinoma (BRCA) (C), and pancreatic adenocarcinoma (PAAD) (D). Abbreviations: TCGA: The Cancer Genome Atlas

**Figure S2**. Expression of coatomer complex subunit beta 2 (COPB2) in various types of cancers. (A) COPB2 showed higher expression in cancer patients than in healthy individuals. (B) Comparison between the expression of COPB2 in colorectal cancer in healthy and primary tumor tissues. (C) Levels of the phosphorylated form of COPB2 in each stage of cancer. Z-values represent standard deviations (SD) from the median across samples for the given cancer type. Abbreviations: CPTAC, Clinical Proteomic Tumor Analysis Consortium; RCC, renal cell carcinoma; UCEC, uterine corpus endometrial carcinoma

**Figure S3**. Expression of xanthine dehydrogenase (XDH) in various types of cancers. (A) XDH showed higher expression in cancer patients than in normal healthy individuals. (B) Expression of XDH in colorectal cancer in normal healthy and primary tumor tissues. (C) Expression of XDH in each stage of cancer. Z-values represent SD from the median across samples for the given cancer type.

**Figure S4**. Expression of formin homology 2 domain-containing protein 1 (FHDC1) in various types of cancers. (A) FHDC1 showed higher expression in cancer patients than in normal healthy individuals. (B) Expression of FHDC1 in colorectal cancer in normal healthy and primary tumor tissues. (C) Expression of FHDC1 in each stage of cancer. Log2 spectral count ratio values from CPTAC were first normalized within each sample profile, and then normalized across samples.

**Figure S5**. Expression of FRAS1-related extracellular matrix 1 (FREM1) in various types of cancers. (A) FREM1 showed higher expression in cancer patients than in normal healthy individuals. (B) Expression of FREM1 in colorectal cancer in normal healthy and primary tumor tissues. (C) Expression of FREM1 in each stage of cancer. Log2 spectral count ratio values from CPTAC were first normalized within each sample profile, and then normalized across samples.
